# Insights into the SARS-CoV-2-Mediated Alteration in the Stress Granule Protein Regulatory Networks in Humans

**DOI:** 10.3390/pathogens10111459

**Published:** 2021-11-11

**Authors:** Kartikay Prasad, Abdullah F. Alasmari, Nemat Ali, Rehan Khan, Adel Alghamdi, Vijay Kumar

**Affiliations:** 1 Amity Institute of Neuropsychology & Neurosciences (AINN), Amity University, Noida 201303, India; kartikayprasad@gmail.com; 2 Department of Pharmacology and Toxicology, College of Pharmacy, King Saud University, P.O. Box 55760, Riyadh 11451, Saudi Arabia; afalasmari@ksu.edu.sa (A.F.A.); nali1@ksu.edu.sa (N.A.); 441105941@student.ksu.edu.sa (A.A.); 3 Department of Pathology, Case Western Reserve University, Cleveland, OH 44106, USA; rehan.khan3@case.edu

**Keywords:** SARS-CoV-2, stress granule proteins, protein–protein interaction, network, drug, miRNAs

## Abstract

The rapidly and constantly evolving coronavirus, SARS-CoV-2, imposes a great threat to human health causing severe lung disease and significant mortality. Cytoplasmic stress granules (SGs) exert anti-viral activities due to their involvement in translation inhibition and innate immune signaling. SARS-CoV-2 sequesters important SG nucleator proteins and impairs SG formation, thus evading the host response for efficient viral replication. However, the significance of SGs in COVID-19 infection remains elusive. In this study, we utilize a protein-protein interaction network approach to systematically dissect the crosstalk of human post-translational regulatory networks governed by SG proteins due to SARS-CoV-2 infection. We uncovered that 116 human SG proteins directly interact with SARS-CoV-2 proteins and are involved in 430 different brain disorders including COVID-19. Further, we performed gene set enrichment analysis to identify the drugs against three important key SG proteins (DYNC1H1, DCTN1, and LMNA) and also looked for potential microRNAs (miRNAs) targeting these proteins. We identified bexarotene as a potential drug molecule and miRNAs, hsa-miR-615-3p, hsa-miR-221-3p, and hsa-miR-124-3p as potential candidates for the treatment of COVID-19 and associated manifestations.

## 1. Introduction

The causative agent of COVID-19, severe acute respiratory syndrome coronavirus 2 (SARS-CoV-2), is an enveloped, single-stranded ~30 kb RNA virus of the family *Coronaviridae* [1]. Viruses hijack the host translation machinery in favor of their needs and accomplish virus growth [2,3]. However, to counteract virus growth, host cells have highly specific stress sensors that trigger antiviral responses by suppressing both host and viral translation. The assembly of stress granules (SGs) is a crucial part of host cell stress responses in response to viral infection.

Stress granules (SGs) are membrane-less organelles that store translationally silent mRNA when the cell undergoes stress to regulate mRNA metabolism [4]. SG assembly and disassembly are tightly regulated during viral infection, often reflecting cellular translation status [3,5,6,7]. Several studies have shown that viral entry can interfere with SG formation [8] through inhibition of post-translational modifications [9], sequestration of SG components such as T cell-restricted intracellular antigen 1 (TIA-1), and Ras GTP activating protein-binding proteins G3BP1/2 [10,11], and formation of stable viral ribonucleoprotein (RNP) complexes with key SG proteins [12]. In the early phase of many viral infections, the presence of viral genomic RNAs (gRNAs) activates protein kinase R (PKR), resulting in eIF2α phosphorylation, mRNA translation inhibition, and the formation of SGs enriched with translation initiation factors such as eIF3b. However, in later infection stages, many viruses instead suppress SG formation or disassemble SGs altogether. The mechanisms underlying this switch, and its physiological function, remain unclear. Dysregulation of SG formation and disassembly is involved in viral infection, cancer, and neurodegeneration [13,14,15,16]. 

Coronaviruses such as mouse hepatitis coronavirus and transmissible gastroenteritis virus were shown to induce SG assembly [17]. It has also been shown that the Zika virus capsid protein hijacks G3BP1 and CAPRIN-1 and inhibits the SG formation and thus promotes viral replication [18]. Several recent works also reported that SARS-CoV-2 nucleocapsid (N) protein undergoes RNA-induced liquid–liquid phase separation (LLPS) for its genome packaging and assembly [19,20,21,22]. The SARS-CoV-2 N protein interacts and sequesters key SG proteins including G3BP which leads to attenuation of SG [23,24,25]. These results demonstrate that virus protein can interact with different SG proteins and partition into liquid phases thus indicating the presence of protein-protein interactions. To date, several SARS-CoV-2 human interactomes have been created which aid in comprehending the viral entry, infection, and disease development mechanisms [23,24,26,27]. Analysis of these networks has revealed commonalities and distinctions based on genes and molecular pathways associated with viral pathogenicity. 

The mechanisms underlying SARS-CoV-2 mediated SG dynamics are crucial to identifying important targetable events in the viral replication cycle. We here employed a network-based system biological framework approach as described previously [28,29,30,31], to investigate the molecular interplay between SARS-CoV-2 proteins and human host SG proteins. We created a brain-specific protein–protein interaction (PPI) network of 116 human SG genes targeted by SARS-CoV-2 reported from previous SARS-CoV-2 interactome studies [23,24,25]. The disease–gene interaction network revealed five key genes linked with the majority of brain-related disorders. The gene set enrichment analysis (GSEA) was studied for the identification of drugs affecting the gene expression of selected SG genes. 

## 2. Results

### 2.1. Interaction Network of SARS-CoV-2 Targeted SG Proteins in the Brain

**Figure 1:**
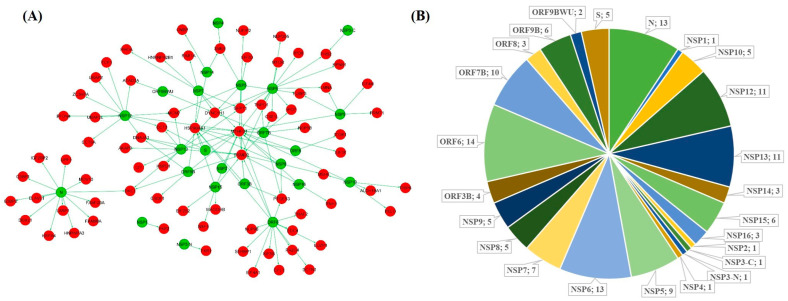
SARS-CoV-2 human interactome. (**A**) Protein–protein interaction network of the 116 stress granule proteins (red) with SARS-CoV-2 proteins (green). (**B**) The number of SG proteins showing interaction with SARS-CoV-2 proteins is represented as a pie chart.

For identifying the SARS-CoV-2 targeted SG proteins, we first retrieved a list of 809 human proteins targeted by viral proteins from three different SARS-CoV-2 interactome studies [23,24,25]. A list of known mammalian SG proteins was retrieved from the MSGP database. A total of 116 SG proteins showing interaction with SARS-CoV-2 proteins were identified by comparing the two lists (Figure 1A). We found that these 116 proteins interact with 22 SARS-CoV-2 proteins with the highest number of interactions to ORF6 (14), N and NSP6 (13), NSP12, and NSP13 (11), ORF7 (10), and NSP7 (7) protein (Figure 1B). 

**Figure 2:**
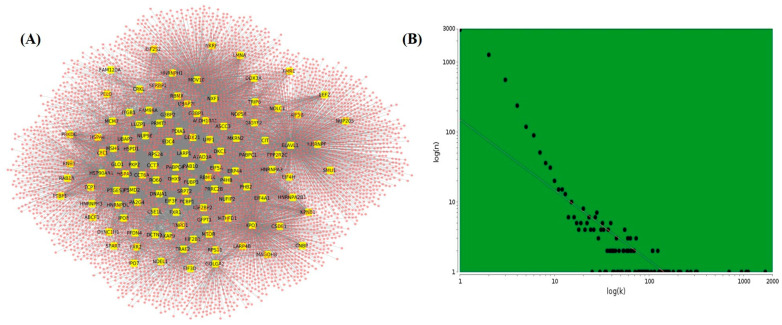
SARS-CoV-2-targeted stress granule genes interaction network in the brain. (**A**) SARS-CoV-2-targeted SG gene (yellow) interaction network in the human brain with neighboring genes (in pink). (**B**) Scatterplot representing the distribution of degree (k) in the SG genes target network.

The PPI network of the brain was retrieved from the TissuevNet2.0 database for preparing the interaction network of SARS-CoV-2 target SG proteins. Using brain PPI, a network of 12,968 proteins with 165,241 interactions was prepared. Further, a subnetwork of 116 identified SG proteins with their direct neighboring protein was made from the brain PPI network. The subnetwork shows 5548 nodes and 13,546 edges (Figure 2A). The subnetwork represents how well connected these 116 identified proteins are in the brain PPI network. The 116 proteins are directly connected with 5432 different proteins in the brain, so any change in the expression of these proteins may have the ability to manipulate the functions of the neighboring proteins directly connected to them. The degree distribution of the network indicated the presence of a scale-free network (Figure 2B). Most of the real-time network follows scale-free property.

### 2.2. Stress Granules-Related Disease–Gene Interaction Network in the Brain

**Figure 3:**
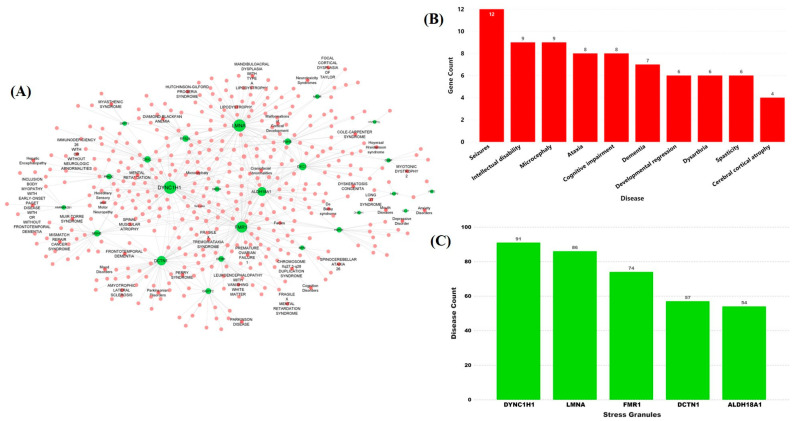
SG gene–disease interaction network. (**A**) Interaction of SG genes and the associated brain diseases. SARS-CoV-2 target SG genes are shown in green, brain-related diseases are represented in pink. (**B**) Bar plot of maximally connected diseases along with the number of SG genes connected to the brain in the disease–gene interaction network. (**C**) Bar plot of key SG genes having maximum connections to various brain diseases in the network.

To understand the role of identified SG genes in the brain-related symptoms in COVID-19 patients, we prepared a disease–gene interaction network. GeneORGANizer and MalaCards databases were used to retrieve the disease–gene-related information for the above-identified 116 SG genes. A gene–disease interaction network was made with 453 nodes and 663 edges (Figure 3A). Four hundred and thirty different brain disorders, including COVID-19, showed interaction with 116 SG genes. The gene–disease interactions displayed many of the disorders that were connected to more than one gene in the network such as seizures (k = 12), intellectual disability (k = 9), microcephaly (k = 9), ataxia (k = 8), cognitive impairment (k = 8), dementia (k = 7), developmental regression (k = 6), dysarthria (k = 6), spasticity (k = 6), and cerebral cortical atrophy (k = 4) (Figure 3B). Similarly, the gene-disease interaction network revealed that many disorders share common genotypes. The network revealed that the majority of the disorders are linked with DYNC1H1 (k = 91), LMNA (k = 86), FMR1 (k = 74), DCTN1 (k = 57), and ALDH18A1 (k = 54) genes and showed interactions with multiple brain disorders (Figure 3C). These genes are thus considered key SG genes. The disease–gene interaction represents the role of SARS-CoV-2 targeting SGs in brain disorders and hence providing a link between COVID-19 and neurological symptoms. It is widely known that the SARS-CoV-2 virus majorly affects the lungs as compared to other parts of the host body [32,33]. We have also prepared a lung/respiratory disease–gene interaction network of the SG genes. The corresponding disease–gene interaction network showed a total of 40 interactions, in which 36 lung/respiratory-affecting disorders were connected with 17 SG genes (Supplementary Figure S1A). The respiratory-related disorders in which the identified SGs play an important role include hypoventilation, respiration insufficiency, aspiration, central hypoventilation, and perry syndrome along with some other syndromes. Interestingly, out of five key SG genes that showed a high number of associations with brain disorders, three genes, namely LMNA (k = 14), DCTN1 (k = 8), and ALDH18A1 (k = 4), also play important roles in disorders having a major impact on lungs and respiratory ability of the patients (Supplementary Figure S1B). 

Targeting these SG genes thus could play significant role in brain as well as lung/respiratory-related disorders and will provide a dual benefit in the process of identifying a potential COVID-19 treatment.

### 2.3. Functional and Pathways Enrichment Analysis of the Selected Genes

**Figure 4:**
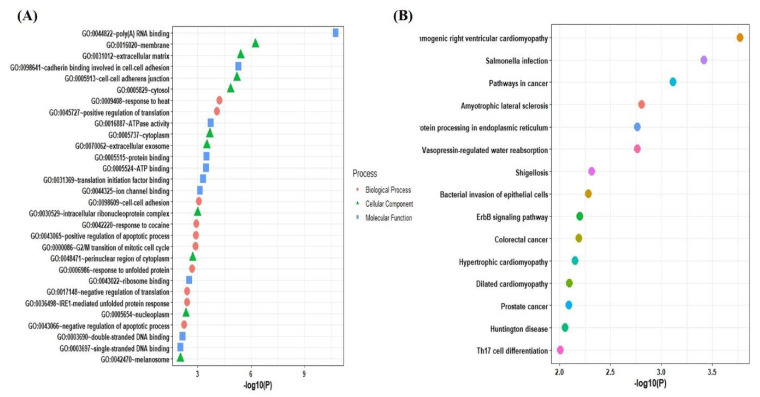
Functional enrichment analysis. (**A**) Gene ontology analysis of 116 SG genes. (**B**) KEGG pathways related to 116 SG genes.

For determining the function and mechanism of the identified SG genes associated with the majority of diseases, a list of these SG genes was submitted to DAVID and Enrichr databases for GO and KEGG pathway analysis. The GO analysis indicated that the biological process was mainly enriched in positive regulation of translation, cell to cell adhesion, positive regulation of the apoptotic process, response to heat, and response to unfolded proteins. The cellular components are significantly enriched in the membrane, extracellular matrix, cell–cell adherens junction, cytosol, and cytoplasm. Molecular functions were mainly enriched in RNA-binding, cadherin binding involved in cell–cell adhesion, ATPase activity, protein binding, ATP binding, and translation initiation factor binding (Figure 4A). According to KEGG pathway analysis, the SG genes participate in the arrhythmogenic right ventricular cardiomyopathy (ARVC) pathway, pathways in cancer, amyotrophic lateral sclerosis pathways, protein processing in the endoplasmic reticulum, and vasopressin-regulated water absorption pathways along with other pathways (Figure 4B). 

### 2.4. GSEA Based Drug Repurposing 

Using the Enrichr web tool, we identified the expression signatures of key SG genes in COVID-19. GSEA of the COVID-19-related gene sets indicated that three genes namely DYNC1H1, LMNA, and DCTN1 were downregulated in human bronchial epithelial cells in COVID-19 after 24hr of infection (GSE17400) (Supplementary Figure S2). Firstly, the DCTN1 gene is also known as Dynactin-1. It is located on chromosome 2p13 and in humans and encodes six different isoforms. The dynactin complex acts as a connector of cargos. It is involved in multiple cellular functions including ER-to-Golgi transport, the centripetal movement of endosomes and lysosomes, chromosomal movements, spindle formation, and axonogenesis. The dysregulation of this gene is known to cause ALS, perry syndrome, neuropathy, distal hereditary motor neuropathy, and other issues related to motor movements [34,35]. Secondly, the LMNA gene is known as Lamin A/C and is a protein-coding gene. Nuclear lamins are the crucial component of the intricate protein mesh that underlies the inner nuclear membrane and confers mainly nuclear and cytosolic rigidity. Lamin proteins are thought to be involved in nuclear stability, chromatin structure, and gene expression. Lamin family proteins make up the matrix and are thought to be evolutionarily conserved. Any dysregulation in the LMNA gene is known to cause Hutchinson–Gilford progeria syndrome, cardiomyopathy, muscular dystrophy, emery-derifusss muscular dystrophy, and lipodystrophy [34,36,37]. The third gene was DYNC1H1, also known as dynein cytoplasmic-1-heavy chain-1. Dyneins are a group of microtubule-activating ATPases that function as molecular motors. They are involved in intracellular motility including retrograde axonal transport, protein sorting, organelle movement, and spindle dynamics. Dysregulation of this gene is known to cause spinal muscular atrophy, Charcot-Marie-Tooth disease, mental retardation, and spinal muscular atrophy [34].

Further, the GSEA of the drug perturbations from GEO database records of downregulated genes revealed bexarotene, also known as targretin, as the top significant enriched candidates showing interaction with the three downregulated genes in COVID-19 (Supplementary Figure S3A). The search in GEO data sets showed that bexarotene in rats upregulated the expression of DYNC1H1, DCTN1, and LMNA genes in the liver, lungs, and mammary glands (Supplementary Figure S3B). 

**Figure 5:**
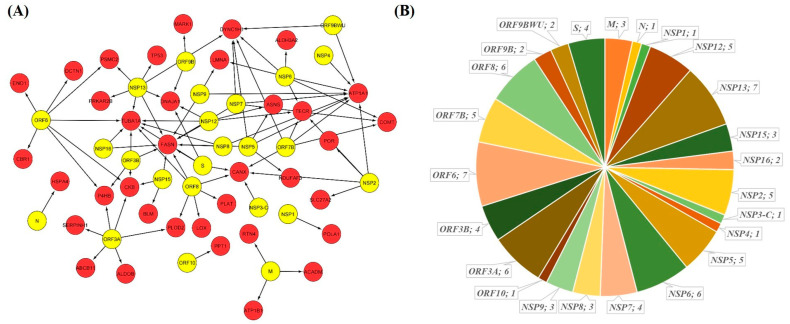
Effect of bexarotene on the SARS-CoV-2 human interactome. (**A**) Bexarotene interacts with 36 (i.e., ~4.4%) of 809 human proteins prey of SARS-CoV-2, with a total of 87 interactions. These 36 proteins potentially interact with 24 (i.e., ~88.8%) of 27 SARS-CoV-2 proteins. (**B**) Drug–gene interaction network of bexarotene and SARS-CoV-2 proteins. The yellow node represents SARS-CoV-2 proteins and the red node represents human proteins targeted by viral proteins in the human host.

Assuming that bexarotene significantly alters the PPI and would inhibit the virus growth, we here studied the drug–protein interactions. Out of a total of 809 human proteins prey of SARS-CoV-2, bexarotene interacts with 36 (i.e., ~4.4%) human proteins and potentially interferes with 24 of 27 (i.e., 89%) SARS-CoV-2 proteins (Figure 5A). The 36 proteins mostly show at least 1–2 interactions with 24 SARS-CoV-2 proteins, totaling 87 interactions (Figure 5B). This finding suggests that bexarotene could be considered as a possible drug for drug repurposing against COVID-19. 

### 2.5. miRNA Based Drug Repurposing

**Figure 6:**
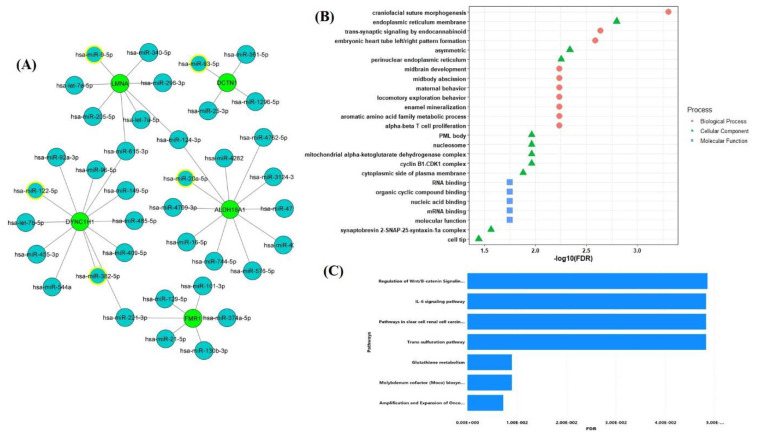
Identification of miRNAs regulating the expression of key five SG genes, (**A**) miRNA-SG genes interaction network. The network displays the miRNAs (blue) targeting five key SG genes (green). The antiviral miRNAs are highlighted with a yellow border. (**B**) Gene Ontology analysis of the antiviral miRNAs interacting with key SG genes. (**C**) KEGG pathways enrichment analysis of antiviral miRNAs.

Apart from chemical-based drug target identification, we also searched for miRNAs as a potential target for the key SG genes. A total of 502,652 miRNA–gene interactions in humans were downloaded from the miRTarBase database. For the top five selected key SG genes, a total of 44 miRNA interactions were identified (Figure 6A). Further, out of the 44 identified miRNAs, we selected miRNAs that have anti-viral properties and identified that out of five key SG genes, four genes interact with at least one antiviral miRNA. DYNC1H1 showed interaction with two antiviral miRNAs—namely has-miR-122-5p and has-miR-382-5p—whereas the other genes LMNA, DCTN1, and ALDH18A1 interacted with has-miR-9-5p, has-miR-93-5p, and has-miR-20a-5p, respectively. ALDH18A1 gene is also known as aldehyde dehydrogenase 18A family member A1 and encodes bifunctional ATP and NADPH mitochondrial enzymes. The protein encoded by this gene reduces glutamate into delta1-pyrroline-5-carboxylate, a critical step in the biosynthesis of proline, ornithine, and arginine. The gene is involved in pathways such as the urea cycle, amino acid synthesis pathways, metabolism pathways, and peptide chain elongation pathways. Dysregulation in this gene is known to cause hyperammonaemia, hyperornithinaemia, hyperargininaemia, and is associated with neurodegeneration, cataract, and connective tissue disease [34,38,39].

Gene ontology enrichment analysis of the identified antiviral miRNAs revealed that the biological process is enriched in craniofacial suture morphogenesis, trans-synaptic signaling by endocannabinoid, embryonic heart tube left/right pattern formation, and alpha-beta cell proliferation. The cellular components were significantly located in the endoplasmic reticulum membrane, asymmetric, perinuclear endoplasmic reticulum, PML body, cyclin B1-CDK1 complex, and nucleosome. The molecular functions were mainly enriched in RNA binding, mRNA binding, nucleic acid binding, and organic cyclic compound binding (Figure 6B). Moreover, the pathway enrichment analysis revealed the role of miRNAs in glutathione metabolism, amplification of expansion of oncogenic pathways as metastatic traits, molybdenum cofactor biosynthesis, IL-6 signaling pathways, pathways in clear cell renal cell carcinoma (ccRCC), trans-sulfuration pathways, and regulation of Wnt/B-catenin signaling pathways (Figure 6C).

## 3. Discussion and Conclusions

The activation of SGs upon viral infection has been considered as a host antiviral mechanism [3,5]. Besides blocking viral gene expression via translation arrest, SGs also eliminate viral factors to inhibit their growth [6,18,40]. Many viruses have developed strategies to disrupt SG formation to help their growth [18,41]. MERS-CoV protein 4a, HCV NS5A, JEV NS2A protein, and Sendai virus C protein target PKR and prevent SG formation [42,43,44,45,46]. Enterovirus (EV 71) protease 3Cpro cleaves G3BP1 and disrupts SGs assembly following EV71 infection [47]. The poliovirus, foot-and-mouth disease virus, and feline calicivirus adopt similar mechanisms to inhibit SG assembly [48,49,50]. Recent studies have shown that the SARS-CoV-2 N protein prevents SG formation by preventing PKR autophosphorylation and activation, and by sequestering G3BP1 [22,51]. These observations indicate a fairly conserved mechanism of escaping the host defense by beta coronaviruses. Several escaping mechanisms from host defense by the SARS-CoV-2 virus have been recently described [29,30,31,52,53], but it is not clear whether SARS-CoV-2 targets host key SG components.

Here, we adopted an integrative network biology approach to decipher the SG genes-based molecular alliance of COVID-19 with neurological disorders. Our findings showed that 116 SG proteins were targeted by 27 SARS-CoV-2 proteins. The results of the PPI network indicate that these SG proteins operate in a highly interconnected network that coordinates many activities of the cellular RNA homeostasis. The brain-specific disease-genes network showed that 430 different brain disorders including COVID-19 interact with 116 SG genes. In this study, diseases such as seizures, intellectual disability, microcephaly, ataxia, cognitive impairment, dementia, developmental regression, and dysarthria represented the most connected diseases based on different SG genes—DYNC1H1, LMNA, FMR1, DCTN1, and ALDH18A1. Next, to repurpose a drug targeting the most common shared SG genes between SARS-CoV-2 and neurological complications, a GSEA analysis was performed. Based on the enrichment analysis, bexarotene was identified as the top significant enriched candidate interacting with the three downregulated SG genes in COVID-19.

Bexarotene (antineoplastic retinoid) is a synthetic high-affinity retinoid X receptor agonist used in the treatment of cutaneous T cell lymphoma, non-small cell lung cancer, and breast cancer [54,55]. Bexarotene also exerts anti-inflammatory effects by downregulating IL-6, IL-8, monocyte chemoattractant protein 1 (MCP-1), and high mobility group box-1 [56]. It has been shown previously that AM580 and tamibarotene belongs to the same drug class as bexarotene, displayed broad-spectrum antiviral activities against influenza viruses, enterovirus A71, Zika virus, adenovirus, MERS-CoV, and SARS-CoV [57]. Recently, Yuan et al. [58] showed that abiraterone acetate and bexarotene effectively inhibit SARS-CoV-2 replication in vitro. Bexarotene has also been shown as a potential drug target of ACE2, TMPRSS2, and AAK1 through bioinformatic analysis [59]. Thus, bexarotene could be regarded as a candidate drug for repurposing in COVID-19.

We also identified three miRNAs (hsa-miR-615-3p, hsa-miR-221-3p, and hsa-miR-124-3p) which target at least two of the five key SG genes. The miRNA, hsa-miR-124-3p, helps in regulating the inflammatory mechanisms in viral infection by targeting cytokine regulating immune expressed genes and associated transcription factors [60]. Moreover, hsa-miR-124-3p was found to be downregulated in JEV-infected human neural stem cells [61]. The miR-124-3p agomir reduced pro-inflammatory cytokines IL-6 and TNF-α levels and thus was able to protect against pulmonary injury [62]. It has been shown that SARS-CoV-2 hijacks Ddx58 which is involved in miRNA biogenesis and mRNA splicing to help its replication. The miRNA, miR-124-3p, can bind to the 3’-UTR of Ddx58 and downregulate the Ddx58. In one study, Arora et al. showed that overexpression of miR-124-3p would degrade the Ddx58 and inhibit the replication of the SARS-CoV- 2 genome [63].

The miRNA, hsamiR-124, has been shown to inhibit influenza and RSV infection by the reduction in mitogen-activated protein kinase-activated protein kinase 2 (MAPKAPK2 or MK2) [64]. Moreover, according to one study, MK2 was predicted to be targeted by miR-615-3p and was reduced in the lungs of COVID-19 patients [65]. The miRNA, hsamiR-221-3p, is found to be upregulated in hamster lung tissue infected with SARS-CoV-2. It targets ADAM17 which is involved in ACE2-dependent shedding linked with lung pathogenesis [66]. 

Our study thus utilizes a comprehensive protein–protein interaction network to map the interplay between SARS-CoV-2 proteins with human SG proteins along with their functional annotations. Therefore, delineating the effect of SARS-CoV-2 infection on human translational regulatory networks is central for identifying effective drug targets against COVID-19. 

## 4. Methods

### 4.1. Identification of SARS-CoV-2 Interacting Human SG Proteins from SARS-CoV-2-Human Interactome 

A list of 809 human target proteins known to interact with SARS-CoV-2 viral proteins was retrieved from three different SARS-CoV-2 interactome studies [23,24,25]. The mammalian stress granules proteome (MSGP) database [67] was used for retrieving the list of SG proteins. The MSGP database curates the information regarding the SGs using published literature available on PubMed and other sources. Further information regarding each SG protein was then obtained from Uniport, GeneDatabase, and OMIM. The database also provides the expression profile of SG proteins in the context of neurodegenerative diseases. A list of 464 SG proteins was obtained from the MSGP database. Out of 809 proteins, a total of 116 SG proteins were identified as known to have direct interaction with SARS-CoV-2 viral proteins.

### 4.2. Protein–Protein Interaction of Identified SG Proteins in the Human Proteome

The human proteome interaction data were obtained from TissueNet v.2 databases [68]. For human PPI, the TissueNet database provides the quantitative tissue association. For preparing an extensive interaction network, protein-based assay profiles and RNA-Seq profiles were gathered from the human protein atlas (HPA) and the genotype tissue expression project (GTEX), respectively. BioGrid, MINT, DIP, and IntAct were the four major databases used for extracting the experimentally validated protein interaction information for the PPI network. A list of 116 identified SG proteins interacting with SARS-CoV-2 proteins was used for creating a subnetwork having interactions between SARS-CoV-2 proteins and SG proteins and the directly connecting first neighbors.

### 4.3. Preparation of Disease–Gene Interaction Network Specific to Brain

After obtaining the interaction network of SARS-CoV-2 target SG proteins and their neighboring proteins in the human proteome, the MalaCards database [69] along with the GeneORGANizer database [70] was used to identify genes playing role in the brain, cerebellum, and head-related disorders. GeneORGANizer allows the user to identify the organs in which the query genes are expressed along with the information related to disorders caused by the query genes in these organs. The database delivers organ-specific gene-disease information from highly curated DisGeNET [71] and human phenotypes ontology (HPO) tools. The MalaCards database scans 74 databases to provide disease–gene relationship information regarding the query genes. Disease–gene interactions were considered for further study if they had HPO identifiers. A total of 1246 disease–gene interactions were obtained, of which 430 different brain disorders including COVID-19 were linked with 18 SG genes. The brain gene–disease interaction network was created using the Cytoscape tool [72].

Similarly, we identified the role of SG genes in lung/respiratory-related disorders by creating a lung disease–gene interaction network. The corresponding lungs/respiratory-related disease–gene interaction network was prepared with a total of 40 interactions, in which 36 different lung/respiratory-affecting disorders were linked with 17 SG genes.

### 4.4. Calculation of Topological Properties of the PPI Network

The topological properties of the network were calculated to identify the top genes showing associations with brain-related disorders through the network analyzer plugin of Cytoscape, similar to our previous studies [28,31]. The calculated network topological properties included degree centrality (k) and betweenness centrality ($C_{b}$) values for identifying the highly connected nodes. Degree centrality (k) indicates the number of interactions made by a node with another node in the network and thus conveys the significance of that node in controlling the network interactions, and is expressed as:

(1) $$\mathrm{Degree}\;\mathrm{centrality}\;(k)=\sum_{a\varepsilon K_{b}}w\left(a,b\right)$$

where, $K_{a}$ is the node set containing all the neighbors of node a, and *w(a,b)* is the weight of the edge between node *a* and node *b*.

The other parameter, betweenness centrality ($C_{b}$), indicates the degree to which nodes occur with each other in the shortest path. A node with higher betweenness centrality denotes stronger control over the information flow in the network. It is expressed as:

(2) $$C_{b}(u)\;=\;\sum_{k\neq u\neq f}\frac{p\left(k,u,f\right)}{p\left(k,f\right)}$$

where, *p(k,u,f)* is the number of interactions between nodes *k* and *f* that passes through *u*, and *p(k,f)* denotes the total number of shortest interactions between node *k* and *f*.

### 4.5. Gene Ontology and Pathway Enrichment Analysis

Next, the enrichment analysis of the PPI network was explored using the DAVID (Database for annotation visualization and integrated discovery) tool [73]. DAVID utilizes the Gene Ontology (GO) and Kyoto Encyclopedia of Genes and Genomes (KEGG) database for studying the functional enrichment of the selected genes. GO analysis includes functional annotation of genes at the biological, molecular, and cellular level. Functions and pathways with *p*-values < 0.05 were considered significantly enriched and included in the results. 

### 4.6. Identification of Drugs through Gene Set Enrichment Analyses (GSEA) Analysis 

Further, to identify the drugs modulating the expression of key SG genes, GSEA was performed through the Enrichr web server, which stores the expression information of almost 200,000 genes from more than 100 gene set libraries [74,75]. The Enrichr database provides multiple drug–gene interaction information along with gene expression profiles obtained from the gene expression omnibus (GEO) database.

### 4.7. Identification of microRNAs as a Gene Expression Regulator

MicroRNAs (miRNAs) are small non-coding RNAs that can regulate the expression of genes by interacting with target messenger RNAs. miRNAs play an important role in many viral diseases such as Ebola, SARs, and HIV by downregulating the host’s genes [76]. These properties make miRNAs a potential therapeutic target. For identifying miRNAs interacting with five key SG genes, different miRNA–gene interaction databases including miRTarBase, miRbase, miRDB, and miRNet2 were screened [77,78,79,80]. A list of miRNAs showing antiviral properties was also retrieved from the VIRmiRNA database [81]. The GeneTrail [82] database was explored for the GO and pathway-based enrichment analysis of the selected antiviral miRNAs. 

## Data Availability

Data is contained within the article or in Supplementary Material.

## Supplementary Materials

The following are available online at https://www.mdpi.com/article/10.3390/pathogens10111459/s1, Figure S1: SG gene disease interaction network; Figure S2: Effects of SARS CoV 2 challenges on the ex-pression of LMNA, DYNC 1 H 1 and DCTN 1 genes; Figure S3: (A) GSEA of the drug perturba-tions from GEO database records of downregulated genes identify bexarotene as potential drug candidate against SARS CoV 2 infection. (B) Bexarotene increasing the expression of LMNA, DYNC1H1, and DCTN1 genes as shown by enriched GEO records (GSE39).
