# Supplementary material for: Insights into the SARS-CoV-2-Mediated Alteration in the Stress Granule Protein Regulatory Networks in Humans
Source: Pathogens. 2021 Nov 11;10(11):1459. doi: 10.3390/pathogens10111459 (PMC8624858; doi:10.3390/pathogens10111459)
Supplement: Supplementary file 1 [file pathogens-10-01459-s001.zip › pathogens-1420999-supplementary.pdf]

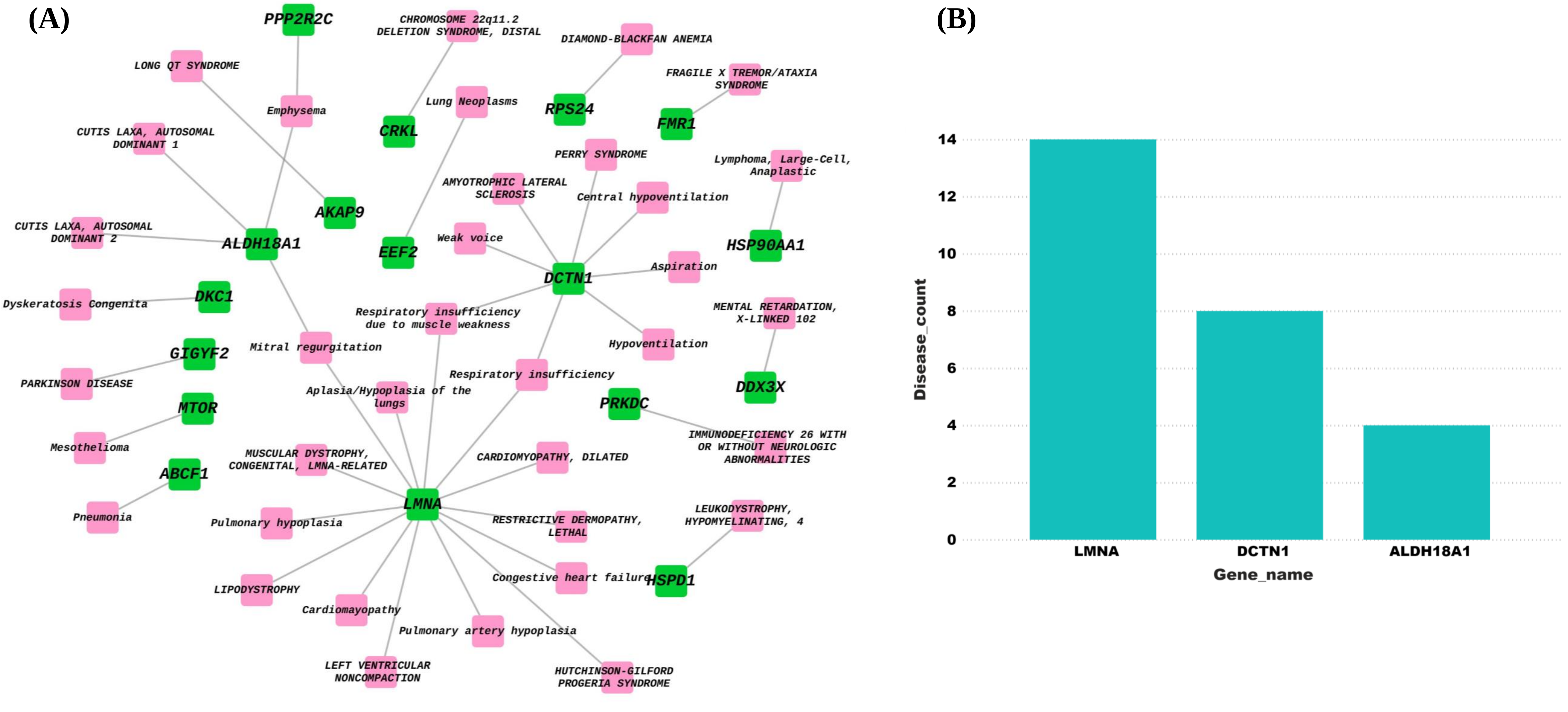

Supplementary Figure 1. SG gene-disease interaction network. (A) Interaction of SG genes and the associated lung/respiratory affecting diseases. SARS-CoV-2 target SG genes are shown in green, lung/respiratory-related affecting diseases are represented in pink. (B) Bar plot of key SG genes having maximum connections to various lung/respiratory affecting diseases in the network.

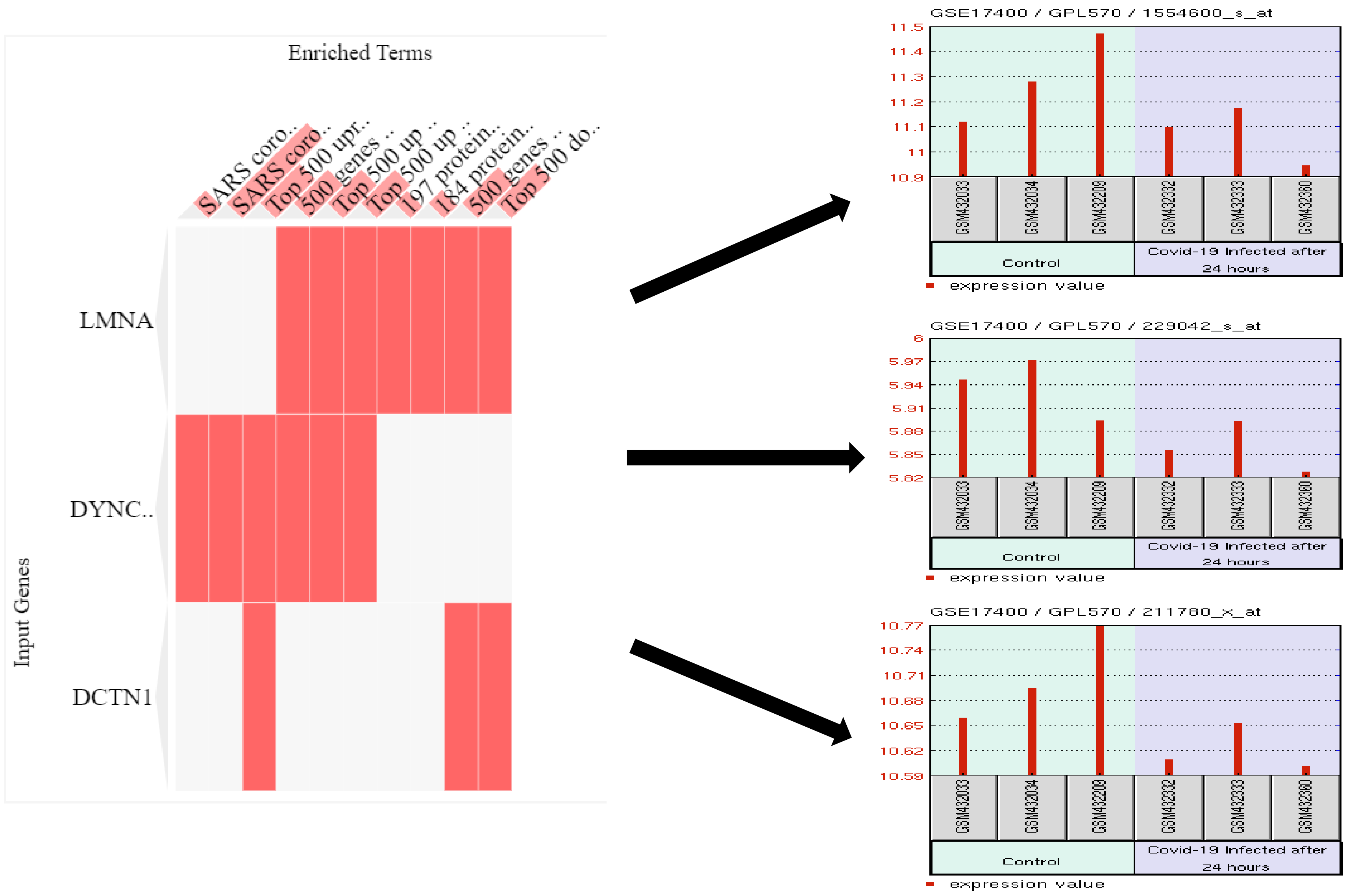

Supplementary Figure 2. Effects of SARS-CoV-2 challenges on the expression of LMNA, DYNC1H1, and DCTN1 genes. (A) GSEA of the COVID-19 related gene sets revealed mostly downregulation of the key SG genes. GEO profiles of the LMNA, DYNC1H1, and DCTN1 genes expression in human bronchial epithelial cell in COVID-19 condition after 24hr of infection (GSE17400).

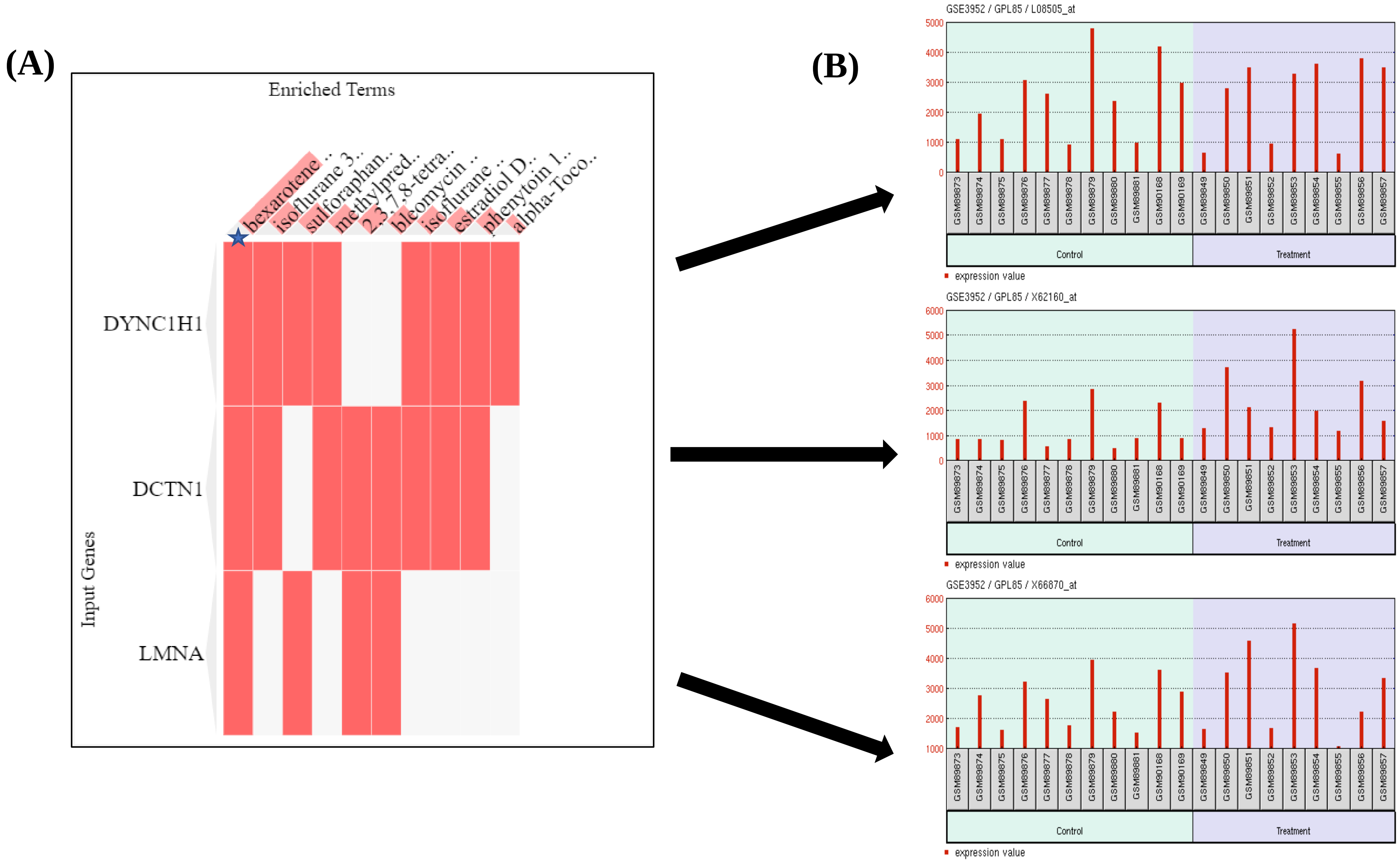

Supplementary Figure 3. (A) GSEA of the drug perturbations from GEO database records of downregulated genes identify bexarotene as potential drug candidate against SARS CoV 2 infection. (B) Bexarotene increasing the expression of LMNA, DYNC1H1, and DCTN1 genes as shown by enriched GEO records (GSE3952).
